# Phase-Consistency-Adaptive Multi-Path Total Focusing Ultrasonic Imaging for Delamination Quantification in L-Shaped CFRP Corner Parts

**DOI:** 10.3390/s26154885

**Published:** 2026-08-03

**Authors:** Jie Ding, Jinming Cao, Tengfei Ma, Haodong Chen, Jun Zhang, Zheng Xu, Jiansheng Jiang, Jingli Yan, Hui Ding

**Affiliations:** 1 The School of Materials Science and Engineering, Southeast University, Nanjing 211189, China; 2 Shanghai Research Institute of Materials Co., Ltd., Shanghai 200437, China; 3 Shanghai Key Laboratory of Engineering Materials Application and Evaluation, Shanghai 200200, China; 4 The School of Power and Mechanical Engineering, Wuhan University, Wuhan 430072, China

**Keywords:** anisotropy, ray tracing, carbon fiber-reinforced polymer (CFRP) composites, total focusing method (TFM), image fusion

## Abstract

The delay-and-sum total focusing method (TFM) for ultrasonic full matrix capture (FMC) depends on accurate ray path and travel time computation. In L-shaped carbon fiber-reinforced polymer (CFRP) corner parts, elastic anisotropy, multilayer stacking, and curvature-induced ray path non-uniqueness generate strong stripe-like coherent clutter (deterministic structural echoes), degrading focusing and sizing. To address this, we search multiple physically plausible candidate ray paths and propose a phase-consistency-adaptive multi-path fusion TFM (PCA-MPF-TFM) that performs pixel-wise path selection and fusion. The method is validated using pulse-echo FMC data acquired with a water-immersion linear array from a 6.4 mm-thick L-shaped CFRP specimen containing three 3 mm-diameter polytetrafluoroethylene (PTFE) inserts; the two within the concave-side inspection region were quantitatively evaluated. Compared with conventional isotropic TFM, an edge-adjacent delamination previously masked by structural noise is consistently detected with a 9.2 dB signal-to-noise ratio (SNR) and a 0.2 mm length error. For the second delamination, the SNR improves by 25 dB and the length error decreases from 0.6 mm to 0.2 mm. Experimental results demonstrate improved defect detectability and noise robustness under curved, anisotropic, and multilayer propagation while maintaining sub-millimeter sizing accuracy.

## 1. Introduction

Carbon fiber-reinforced polymer (CFRP), featuring high specific strength and excellent corrosion resistance, has become an indispensable strategic material in the modern aerospace industry [1]. With rapid advances in manufacturing processes, CFRP applications have expanded from secondary load-bearing components such as interior parts and fairings to primary load-bearing structures such as fuselage keels, wing spars, and center wing boxes, and have been widely adopted in various unmanned aerial vehicles and spacecraft [2,3]. Nonetheless, in complex joints and corner geometries, such as L-shaped or C-shaped corner parts, the occurrence of inter-ply defects, including fiber waviness, local resin-rich regions, and porosity, remains a possibility [4]. Under load conditions, they may grow and trigger inter-ply delamination, which markedly degrades the mechanical performance and structural integrity of CFRP [5]. Therefore, a nondestructive evaluation technique that can effectively detect and quantify internal defects is urgently required to ensure the reliability of safety-critical structures.

Phased-array ultrasonic testing, offering strong penetration capability and practical deployability, has become a mainstream approach for internal-defect inspection in composite structures [6,7,8]. Compared with conventional single-element ultrasonics, beam steering and dynamic focusing are enabled through electronic control, which improves scan efficiency and accuracy [9]. Nevertheless, inspection of geometrically complex L-shaped CFRP corner components, such as corner connectors in aircraft structures, remains challenging. Compared with CFRP plates, wave propagation in corner regions is governed by anisotropic fiber layup and multilayer stacking, and is further modulated by curvature-induced circumferential gradient effects [10]. This coupling increases propagation complexity and renders true ray paths difficult to predict accurately.

To address the complexity of propagation paths caused by anisotropy and curvature coupling, engineering practice often employs curved [11] or flexible array probes that conform to the component surface [12], enabling ultrasonic waves to propagate primarily along the thickness direction. Since each ply typically exhibits nearly uniform wave velocity and acoustic impedance in the thickness direction, this perpendicular incidence method can significantly mitigate the impact of layered inhomogeneities on beam propagation [13]. However, relying solely on normal incidence fails to provide sufficient angle diversity, and the effective focusing region of electronically steered beams is limited [14,15,16]. Consequently, more research is focusing on post-processing imaging techniques.

The Total Focusing Method (TFM), which performs post-processing on data acquired via full matrix capture (FMC), is widely regarded as the “gold standard” in ultrasonic imaging due to its high resolution and robustness [17,18]. However, these advantages are accompanied by a substantial computational burden. For a N element array and an imaging domain containing (P) pixels, conventional time-domain TFM evaluates the delay-and-sum contributions of all ($N^{2}$) transmit–receive pairs at every pixel, resulting in a computational workload that scales approximately as $\left(O\left(PN^{2}\right)\right)$. The processing cost therefore increases rapidly with the number of array elements and the imaging-grid density, while the storage and transfer requirements of FMC data also increase with the number of transmit–receive channels and temporal samples. These requirements can hinder real-time or high-throughput implementation unless parallel or hardware-accelerated computation is employed [14,19]. The core of TFM lies in enforcing a delay law to achieve point-by-point focusing over the region of interest, and the accuracy of this delay law depends directly on reliable time-of-flight prediction and acoustic path modeling. Conventional ray tracing typically adopts iterative solutions based on Snell’s law to determine reflection and refraction of the beam at interfaces [19]; However, for components exhibiting complex acoustic characteristics and intricate structural architectures, the internal wave propagation becomes highly complicated, making accurate computation extremely challenging [20,21,22], particularly, in the curved corner regions of CFRP components, the coupled effects of geometric curvature, material anisotropy, and laminated architecture substantially complicate the solution process, rendering the propagation mechanisms within the radius zone (R-zone) difficult to elucidate [23,24]. To balance computational efficiency with practical implementability, a more pragmatic approach is to construct a discretized model capable of representing key material characteristics and to replace traditional ray tracing with shortest-path search algorithms from computational graph theory [25]. The effectiveness of such methods is mainly constrained by two factors: (i) whether the discretized model can adequately capture the material’s spatial anisotropy and interlaminar structural features, and (ii) whether the candidate paths can closely approximate the true wave propagation trajectory.

To address the above issues, existing approaches can be broadly categorized into two categories. The first category, typically targeting relatively low-frequency ultrasonic inspection, treats multidirectional corner layups as an equivalent homogeneous anisotropic medium [26]. The corner region is discretized in the circumferential and through-thickness directions, and an angle-dependent velocity model is established experimentally to characterize the effective anisotropy methods such as the Back-Wall Reflection Method (BRM) and Through Transmission Method (TTM) [27]. Despite the homogenization method reducing modeling and computational complexity, when the single-layer thickness of carbon fiber plates is on the same order of magnitude as the ultrasonic wavelength, neglecting interlayer refraction effects introduces non-negligible propagation delay errors [28]. The second group regards the corner as an anisotropic heterogeneous medium, discretizes the domain into sector-shaped micro-elements, and assumes an approximately constant fiber orientation within each element, thereby more faithfully capturing circumferential gradient effects and the features associated with laminated stacking [29,30].

Although these two categories differ in their discretized modeling strategies, they generally approximate the true propagation path by the path of minimum travel time. This approximation is often acceptable for planar components; however, in curved corner regions, the coupled effects of curvature, anisotropy, and layered architecture may give rise to multiple candidate ray paths that satisfy Snell’s law locally. As a result, the actual propagation path does not necessarily correspond to the unique minimum-time path [23].

From a wave-theoretic perspective, Fermat’s principle in its rigorous form states that acoustic propagation follows trajectories for which the travel time functional is stationary [31] (i.e., it attains an extremum or a saddle point), rather than necessarily the minimum-time path. In other words, when the medium exhibits complex acoustic behavior, interfaces are nonplanar, and the solution may be non-unique, the degenerate assumption that the true propagation path coincides with the shortest travel time path no longer necessarily holds true [23]. Luo et al. have further leveraged finite-element simulations of corner regions to extract both the minimum-time paths and alternative candidate paths associated with stronger energy transmission for image reconstruction [32]. The results indicate that, in certain areas, reconstructions guided by the higher-energy paths can yield improved imaging quality compared with those relying solely on the minimum-time assumption. Therefore, to address the local non-uniqueness of acoustic paths in corner regions, it is necessary to investigate pixel-level multi-path selection and fused imaging based on multiple classes of candidate paths that satisfy Fermat’s principle. Experimental investigation of this problem further requires a stable and geometrically well-defined coupling path over the curved inspection surface, so that the effects of anisotropy, layered propagation, and ray path non-uniqueness can be evaluated without being obscured by uncontrolled coupling variations.

Water-coupled pulse-echo phased-array inspection can be implemented through either full immersion or localized water-column coupling. Full immersion provides stable and repeatable acoustic coupling for components that can be accommodated in an immersion tank, whereas robotic squirter or captive-water-column systems allow large-area or geometrically constrained components to be scanned without immersing the entire structure [33,34]. Accordingly, the present study employs water-immersion pulse-echo FMC using quasi-longitudinal bulk waves as a controlled validation configuration. The well-defined water path provides repeatable probe positioning, stable coupling, and a known coupling-layer geometry, thereby enabling the effects of curvature, laminate anisotropy, multilayer stacking, and multi-path propagation on TFM reconstruction to be investigated under controlled conditions. Because the coupling layer is represented separately from the anisotropic CFRP laminate in the travel time model, the proposed framework can be adapted to localized water-column or squirter configurations by updating the coupling-layer geometry and probe standoff, although configuration-specific calibration and experimental validation remain necessary.

Within this controlled water-coupled validation framework, the objective of this work is to develop an adaptive multi-path TFM imaging method for multidirectional CFRP corners under concave incidence, with the aim of improving imaging quality while maintaining high defect-sizing accuracy. The main contributions of this work are as follows: First, a discretized ray tracing model is established by introducing a local coordinate system and imposing a maximum propagation-angle constraint, thereby capturing both circumferential-gradient–induced anisotropic variation and layered microstructure. Second, on this model, Dijkstra-based search is used to efficiently compute multiple candidate propagation paths in curved media. Third, to mitigate ray path non-uniqueness caused by coexisting Snell law solutions at nonplanar interfaces, a pixel-wise focusing-consistency criterion is formulated to select and fuse candidate paths. Finally, the method is validated on full matrix capture data from an L-shaped CFRP corner specimen with embedded delaminations, and its effectiveness and robustness are quantified using multiple metrics.

## 2. Methodology

**Figure 1:**
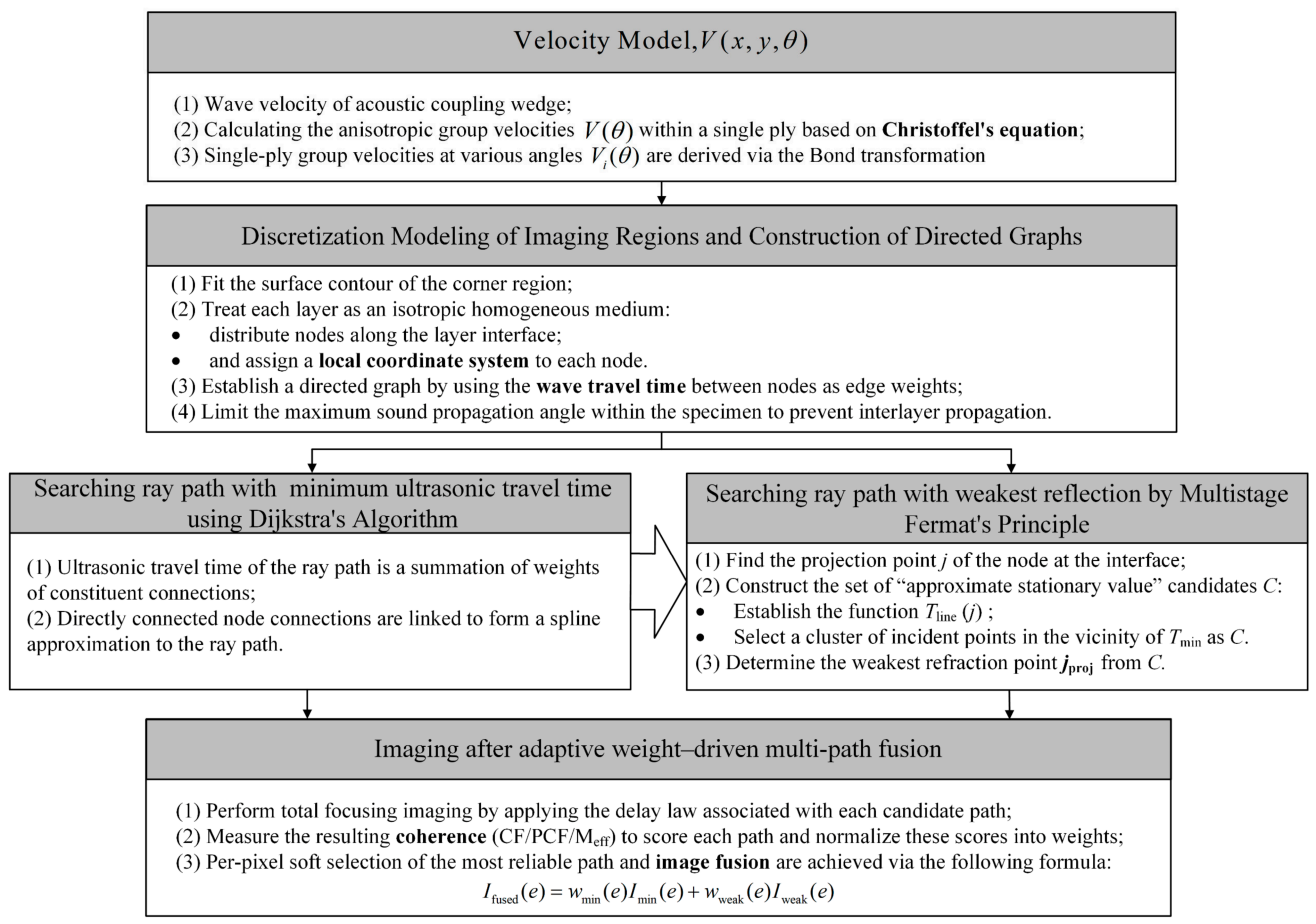
Flowchart of the imaging procedure for the corner region of CFRP using a phase-consistency-driven adaptive multi-path fusion total focusing algorithm.

The main workflow for detecting layered defects in the corner region of an L-shaped CFRP specimen using the phase-consistency-adaptive-driven multi-path fusion ray tracing total focusing method (PCA-MPF-TFM) proposed in this work is shown in Figure 1. Given the known layup, an angle-dependent quasi-longitudinal (qP) group-velocity model is first established for each ply. A curved layered grid is then constructed with local coordinate systems, on which a directed graph is built to compute travel times. For each pixel, two candidate delay hypotheses are generated, namely the minimum travel time path and the weak-refraction (WR) path. Finally, a pixel-wise phase consistency score is used to adaptively weight and fuse the corresponding TFM outputs.

### 2.1. The Group Velocity Model of Corner Part

Each unidirectional ply is modeled as a homogeneous transversely isotropic lamina [35]. In the material coordinate system, its stiffness matrix in Voigt notation is denoted by *C* and given in (1). For a ply with an in-plane orientation angle *φ*, the stiffness tensor is obtained via the standard rotation transformation.

(1) $$C=\left(\begin{array}{cccccc} C_{11} & C_{12} & C_{12} & 0 & 0 & 0\\ C_{12} & C_{22} & C_{22}-2C_{44} & 0 & 0 & 0\\ C_{12} & C_{22}-2C_{44} & C_{22} & 0 & 0 & 0\\ 0 & 0 & 0 & C_{44} & 0 & 0\\ 0 & 0 & 0 & 0 & C_{55} & 0\\ 0 & 0 & 0 & 0 & 0 & C_{55} \end{array}\right).$$

**Figure 2:**
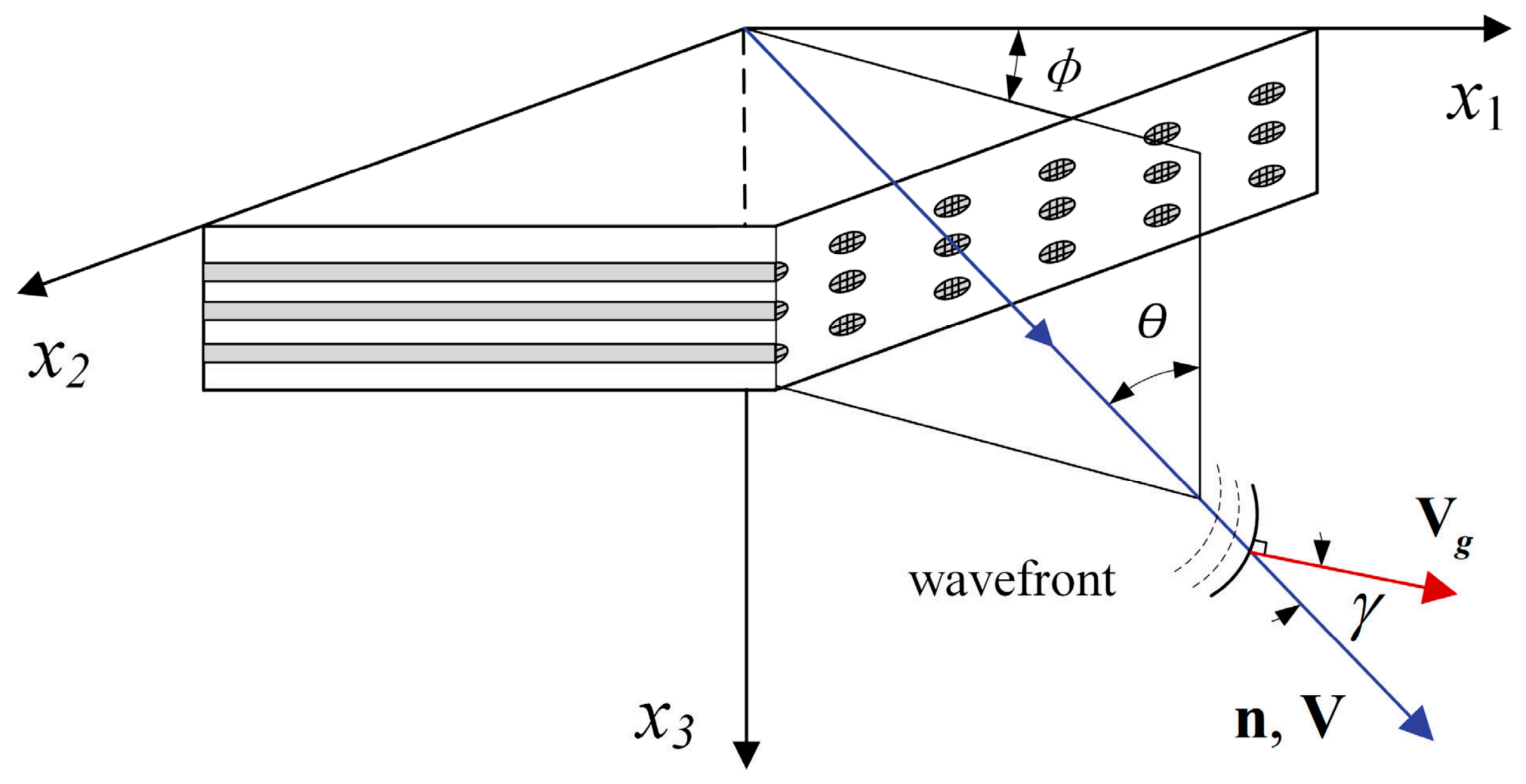
Group and phase velocities. The phase velocity vector **V** is parallel to the normal **n** of the wavefront, while the group velocity **V_g_** represents the propagation of the wave energy and is parallel to the beam orientation.

As illustrated in Figure 2, a propagation direction **n** is parameterized as:

(2) $$n=\left(\sin\theta \cos\phi ,\;\sin\theta \sin\phi ,\;\cos\theta \right).$$

For the quasi-longitudinal (qP) mode, the phase velocity V and polarization vector p are obtained by solving the Christoffel eigenproblem [36]:

(3) $$|C_{ijmn}n_{j}n_{n}-\rho V^{2}\delta_{im}|=0.$$

In anisotropic media, the ultrasonic energy propagates along the group-velocity vector **V*_g_***, i.e., the actual beam direction, which generally deviates from the phase-velocity direction. With the qP-wave polarization p obtained from (3), the i-th component of the group velocity is computed as

(4) $$V_{g,i}=\frac{C_{ijmn}n_{j}p_{m}p_{n}}{\rho V}.$$

Here $C_{\mathrm{ijlm}}$ is the fourth-order stiffness tensor i,j,m,n=1,2,3, ρ is the density, $\delta_{im}$ is the Kronecker delta, and $n_{j}$ and $p_{m}$ denote the components of the propagation direction n and polarization vector p, respectively.

### 2.2. Discretization and Construction of Directed Graphs

To represent the elastic heterogeneity of a curved, layered specimen, the inspection domain is discretized along the surface-normal direction according to ply interfaces, and a local coordinate system is assigned to each grid node. The transversely isotropic representation used here should be regarded as a local engineering approximation for the curved laminate. The concave geometry and ply-wise material orientations are retained through the surface-normal discretization and local coordinate systems, whereas sub-ply heterogeneity and local fiber waviness are not explicitly modeled.

**Figure 3:**
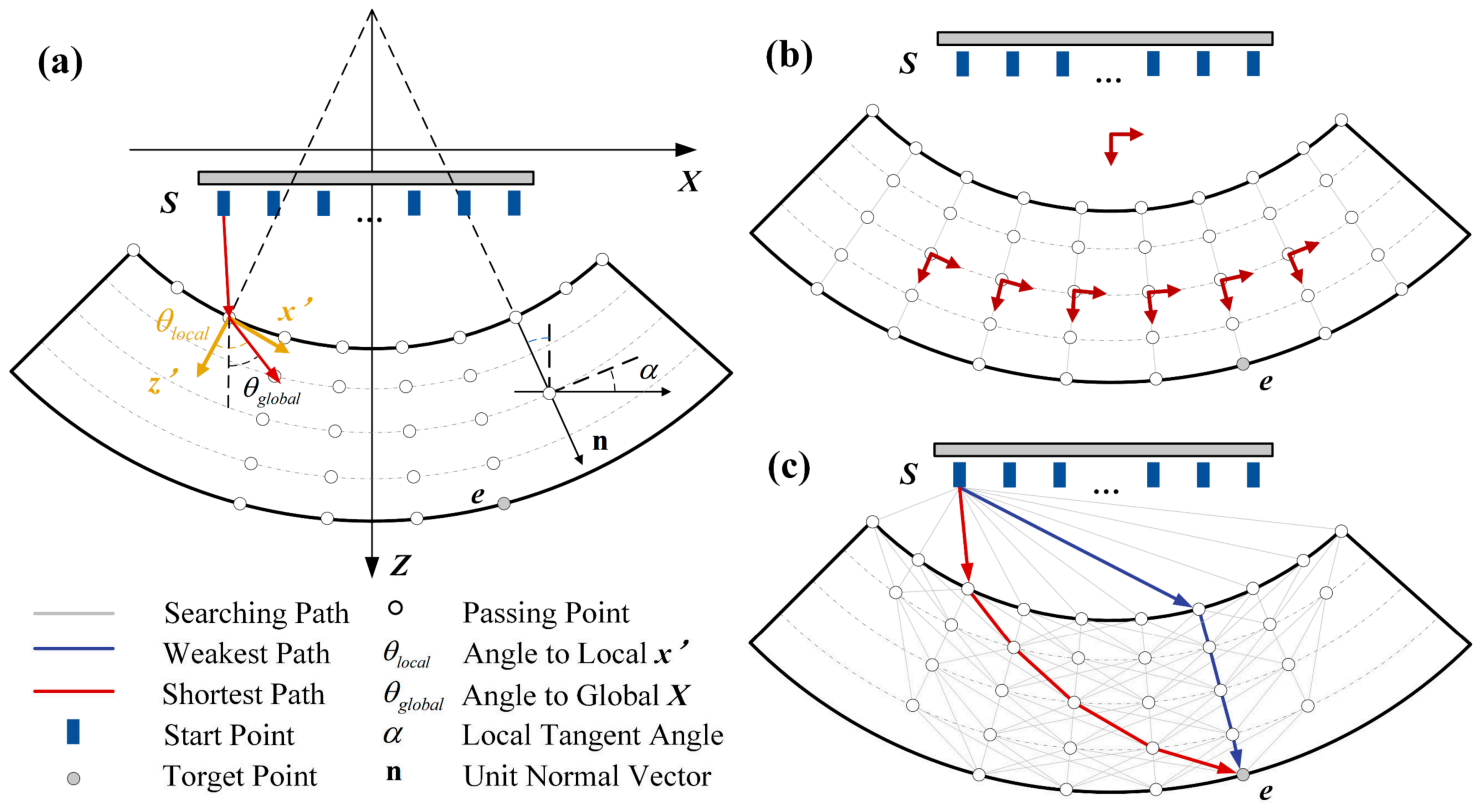
Schematics of the ray tracing method for corner part by shortest path and weak path: (**a**) local coordinate system; (**b**) discretization model; (**c**) ray path finding. *S* denotes the start points, and *e* denotes the target point.

The concave surface is uniformly sampled and fitted to obtain the surface profile $z_{surf}=P(x)$. As shown in Figure 3a, the local tangent angle α is computed from $P^{’}(x)$, and the corresponding unit normal vector is constructed as $n=\left(sin\alpha ,cos\alpha \right)$, ply thicknesses are accumulated along the normal direction to form a depth sequence $\left\{d_{l}\right\}$. The coordinates of nodes on the l-th interface can be expressed as:

(5) $$X_{i}(x)=x+d_{i}\left(\sin\alpha \right),\;Z_{i}(x)=z_{\mathrm{surf}}+d_{i}\left(\cos\alpha \right).$$

Accordingly, a curved layered mesh (*X*, *Z*) of size $N_{z}\times N_{x}$ is obtained. A node-wise local coordinate system is further defined with its local z-axis aligned with the unit normal. The associated rotation angle at node i, denoted by $\alpha_{rot,i}$, is used for subsequent propagation-angle conversion, as shown in Figure 3b.

Based on the discretized mesh, a directed graph *G* is constructed with the following rules.

(1) For each node, directed edges are created only to nodes in the next row, while lateral connections within the same interface are prohibited, ensuring a unidirectional propagation topology from the surface into the interior;
(2) The maximum spreading angle $\theta_{max}$ of the ray in the local coordinate system is constrained to prevent physically implausible cross-layer ray paths. In this study, the maximum admissible local propagation angle was set to $\theta_{max}=70^{\circ }$;

(3) The edge weight is defined as the travel time between adjacent nodes. For a node $i=(x_{i},z_{i})$ and a candidate node $j=(x_{j},z_{j})$, the Euclidean distance is $\Delta s_{ij}$, and the travel time is (6).

(6) $$t_{ij}=\frac{\Delta s_{ij}}{V_{\mathrm{eff}}(p_{j},\;\theta_{\mathrm{local},ij})}.$$

where $p_{j}$ is the layer index of node j (p=0: coupling layer; p≥1: p-th ply), and $\theta_{\mathrm{local},ij}$ is the local propagation direction angle from node i to node j. As shown in Figure 3a, to account for rotation of material principal axes, the propagation angle is first defined in the global coordinate system as:

(7) $$\theta_{\mathrm{global},ij}=\arccos\left(\frac{x_{j}-x_{i}}{\sqrt{{\left(x_{j}-x_{i}\right)}^{2}+{\left(z_{j}-z_{i}\right)}^{2}}}\right)$$

and then transformed into the local coordinate system using α at node i, it can be written as (8):

(8) $$\theta_{\mathrm{local},ij}=\;|\theta_{\mathrm{global},ij}-\alpha |.$$

Accordingly, the effective velocity is defined in a piecewise manner: for node j located in the coupling layer, a constant velocity *V_c_* is used; for node j inside a curve unidirectional CFRP ply, the velocity is obtained from the angle–velocity relationship table of that ply, and can be expressed as:

(9) $$V_{\mathrm{eff}}(p_{j},\theta_{\mathrm{local},ij})=\left\{\begin{array}{ll} V_{c}, & p_{j}=0,\\ V^{\left(p_{j}\right)}(\theta_{\mathrm{local},ij}), & p_{j}\geq 1. \end{array}\right.$$

### 2.3. Candidate Path Tracing and Imaging Under Fermat’s Stationary-Time Principle

TFM is based on delay-and-sum beamforming; the amplitude at an arbitrary focal point can be expressed as:

(10) $$I(x,z)=\left|\sum_{i=1}^{N}\sum_{j=1}^{N}s_{i,j}(t_{i}(x,z)+t_{j}(x,z))\right|.$$

*N* denote numbers of elements. The time delays $t_{i}(x,z)$ and $t_{j}(x,z)$ are the ultrasonic wave travel times from element *i* and *j* to image point (x, y), respectively.

The accuracy of travel time prediction is directly proportional to the quality of imaging. In isotropic media, propagation is often approximated as straight-line travel, and the corresponding travel time can be obtained from simple geometric relations. However, in CFRP corner regions, the coexistence of curved geometry and anisotropic, layered microstructure invalidates the straight-ray assumption. To balance computational efficiency and prediction accuracy, travel times are therefore computed by tracing propagation paths within a discretized model, consistent with Fermat’s stationary-time principle.

Moreover, when ultrasound traverses a nonplanar interface, multiple ray solutions that satisfy Snell’s law may coexist, as illustrated in Figure 3c. Prior studies have shown that, among these candidates, both the minimum travel time path and the weak-refraction path can lead to favorable focusing in different spatial regions [23,32]. Accordingly, the travel time fields corresponding to these two path classes are computed separately and used to reconstruct two respective TFM images, which serve as the basis for the subsequent adaptive fusion strategy.

#### 2.3.1. Global Minimum Travel Time Path Search via Dijkstra’s Algorithm

Applying Dijkstra’s algorithm to a pre-established directed graph *G*, with the element designated as the source node *s*, efficiently computes the shortest travel time to all destination nodes *e*.

In addition, the propagation path can be reconstructed by backtracking via the predecessor array. The steps of the algorithm are detailed in [37] as Combined Formulas (6)–(9); travel time is governed by two factors: geometric spacing between nodes and locally corrected anisotropic velocity. Consequently, the shortest propagation path obtained by Dijkstra’s algorithm on a directed graph fundamentally corresponds to the globally shortest acoustically propagated path that satisfies both propagation topology constraints and maximum diffusion angle constraints.

#### 2.3.2. Weak-Refraction Entry Selection on Concave Surface

A weak-refraction path is defined as a propagation path that minimizes refraction at the concave corner surface and ply interfaces, and thus typically reduces refraction-induced energy loss. Based on the multistage Fermat’s principle, the travel time from an element s to a pixel e can be expressed with respect to a function of the concave surface entry node k:

(11) $$T_{s,e}(k)=T_{s\to k}^{\mathrm{Couple}}+T_{k\to e}^{\mathrm{CFRP}},\;k\in \Gamma_{c},$$

where k denotes the discrete index of a candidate entry node on the concave coupling-layer/specimen interface $\Gamma_{c}$. For a fixed element–pixel pair (s,e), evaluating $T_{s,e}(k)$ along the concave surface converts the entry-point selection into a one-dimensional search problem.

The key to weak-refraction ray tracing lies in a stable selection of the weak-refraction entry point on the curved interface. To this end, we introduce a geometry-projection–constrained local stationary search at the coupling-layer/specimen interface.

**Figure 4:**
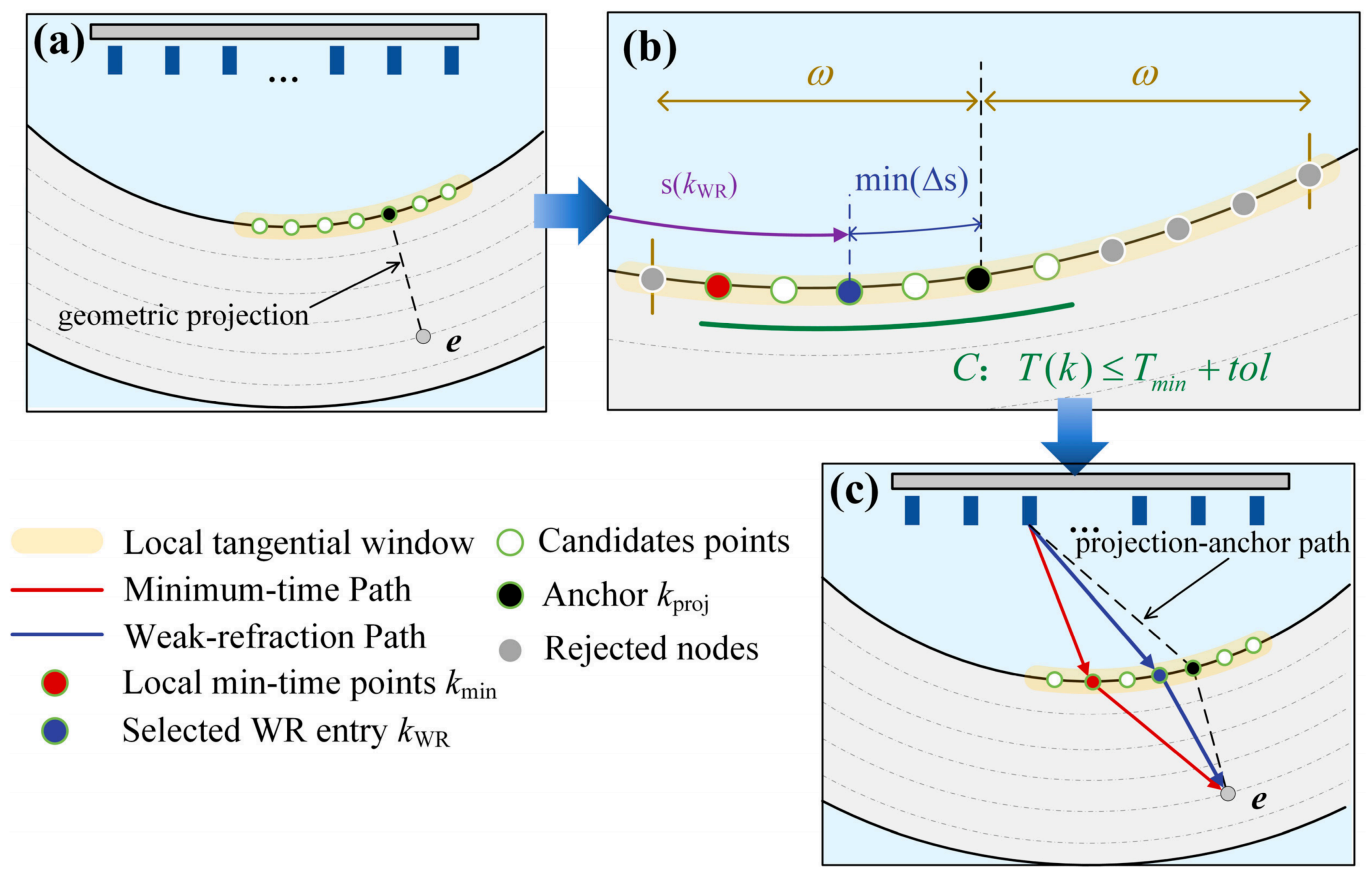
Acoustic-ray schematic of adaptive weak-refraction entry selection on the concave inspection surface: (**a**) determination of the geometric projection anchor *k*_proj_ and local search window; (**b**) construction of the near-stationary candidate set *C* using the time-tolerance criterion and selection of the WR entry *k*_WR_ by the minimum arc-length offset Δs from *k*_proj_; (**c**) comparison of the resulting WR path with the minimum-time path and the projection-anchor path for the same element–pixel pair.

First, the geometric projection of e onto the curved surface is computed, and the nearest surface node is taken as the anchor index *k*_proj_ as shown in Figure 4a. This point corresponds to a straight-ray entry under an isotropic approximation and typically provides higher transmission energy and more reliable channels; therefore, it serves as the search anchor to avoid selecting globally minimum-time solutions that may correspond to severely deviated yet energy-deficient paths.

Secondly, as illustrated in Figure 4b, a local tangential window W(*k*_proj_) centered at *k*_proj_ is defined with half-width ω, namely $\left[k_{proj}-\omega ,k_{proj}+\omega \right]$,. Within this window, the local minimum-time node $k_{min}$ is identified as:

(12) $$k_{\min}=\arg\min_{k\in W(k_{\mathrm{proj}})}T_{s,e}(k),\;T_{\min}=T_{s,e}(k_{\min}).$$

Since the physically realized path in curved scenarios is often associated with a near-stationary region rather than a strictly unique minimum, a time tolerance tol is introduced to retain near-stationary multi-solution candidates while excluding strongly deviated solutions. The candidate set is defined by *C*$=\{k\;|\;T(k)\leq T_{min}+\mathrm{tol}\;\}$ and $tol=\;|T_{\min}|\;\times \;tol_{\mathrm{frac}}$, thereby preserving multiple near-stationary solutions while discarding evidently unreasonable strongly deflected paths. The final WR entry $k_{WR}$ is selected from *C* by minimizing the arc-length offset $\Delta s(k)=|s(k)-s(k_{\mathrm{proj}})|$. The resulting WR path is compared with the minimum-time and projection-anchor paths in Figure 4c. The baseline values used in the WR entry selection were $tol_{frac}=0.02$ and ω=7 surface points, corresponding to an arc-length half-width of approximately 0.362 mm along the concave interface. The local window ω confines the search to the neighborhood of $k_{proj}$, whereas $tol_{fac}$ controls the near-stationary candidate set *C*.

This strategy provides a balance between the global minimum-time path and the geometric projection path (corresponding to high transmission energy), yielding a propagation path that exhibits weak-refraction behavior without violating the constraint implied by Fermat’s principle.

### 2.4. Phase-Consistency-Adaptive Multi-Path Fusion and Imaging

When ultrasound propagates across a nonplanar interface, multiple ray path solutions satisfying Snell’s law may coexist, making it difficult to determine the effective propagation path solely from geometrical-acoustics rules. In corner inspections, different path hypotheses can yield superior focusing in different spatial regions, suggesting that the effective path is spatially variant [23,32]. Therefore, for each pixel *e*, this work considers two candidate path sets, namely the minimum travel time set $P_{min}$ and the weak-refraction set $P_{weak}$, and introduces a pixel-wise criterion that directly reflects focusing correctness for path selection and fusion.

For coherent summation imaging, such a criterion is naturally linked to inter-channel phase consistency [38]; when the assumed delays match the effective propagation, defect-scattered echoes tend to exhibit higher phase alignment across the aperture, whereas structural echoes and multi-interface reverberations generally show larger phase dispersion [39]. Accordingly, a composite ${score}_{p}\left(e\right)$ is constructed for each candidate path $p^{*}\left(e\right)\;\in \;P_{\min}⋃P_{\mathrm{weak}}$ by jointly incorporating the amplitude coherence factor (CF) [40], weighted phase coherence factor (WPCF) and an effective number of channels factor ($M_{eff}$):

(13) $${\mathrm{score}}_{p}(e)={\mathrm{CF}}_{p}(e)\cdot {\mathrm{WPCF}}_{p}(e)\cdot \left(\frac{M_{\mathrm{eff},p}(e)}{K}\right).$$

*K* represents the effective number of channels involved in path selection scoring at pixel e.

A softmax-like normalization is then applied to obtain the fusion weight:

(14) $$w_{\min}(e)=\frac{{\mathrm{score}}_{\min}(e)}{{\mathrm{score}}_{\min}(e)+{\mathrm{score}}_{\mathrm{weak}}(e)+\varepsilon }.$$

*ε* is a small positive regularization constant used in Equations (14), (17) and (20) to prevent division by zero. The signal amplitude weighted by the phase coherence factor is expressed as:

(15) $${\tilde{I}}_{p}(e)={\mathrm{CF}}_{p}(e){\mathrm{PCF}}_{p}(e)I_{p}(e).$$

Combining Equations (14) and (15), the amplitude of pixel *e* after adaptive path fusion can be defined as:

(16) $$I_{fusion}(e)=w_{min}{\tilde{I}}_{min}(e)+(1-w_{\min}){\tilde{I}}_{weak}(e).$$

The three factors play complementary roles. CF measures amplitude coherence after delay compensation but can be biased by high-amplitude yet phase-dispersed echoes under an incorrect delay model. WPCF quantifies phase consistency while using amplitude-based weights to suppress phase estimates dominated by low-amplitude noise or poor coupling. The effective number of channels factor $M_{eff}$, computed via the Kish effective sample size, explicitly accounts for spatially varying numbers of reliable channels and thus stabilizes coherence evaluation when the available aperture changes.

Together, these terms provide a pixel-wise, data-driven mechanism to discriminate and fuse competing ray paths, rather than enforcing a hard path decision a priori. The following provides definitions of the coherence factor and effective channel number factor involved in the method.

#### 2.4.1. Coherence Factor (CF)

The amplitude coherence of multi-channel data after delay compensation is defined in (17).

(17) $${\mathrm{CF}}_{p}(e)=\frac{\left|\sum_{k\in K_{p}(e)}x_{k}^{\left(p\right)}(e)\right|}{\sum_{k\in K_{p}(e)}\left|x_{k}^{\left(p\right)}(e)\right|+\varepsilon }.$$

$x_{k}^{\left(p\right)}(e)$ denotes a complex sample at pixel e and time instant $t_{ij}$. Taking path p as an example, if the full-channel time-domain signal is denoted by $s_{k}(t)$, then $x_{k}^{\left(p\right)}(e)$ is defined as

(18) $$x_{k}^{\left(p\right)}\left(e\right)=s_{k}\left(t_{ij}^{\left(p\right)}(e)\right).$$

#### 2.4.2. Weight Phase Coherence Factor (WPCF)

Based on the phase coherence factor (PCF) [41], WPCF quantifies inter-channel phase alignment while attenuating phase estimates dominated by weak-amplitude noise or poor coupling. This is achieved by introducing amplitude-based weights $w_{k}^{\left(p\right)}(e)$ for each channel; WPCF is defined as:

(19) $${\mathrm{WPCF}}_{p}(e)=\frac{\left|\sum_{k\in \Omega_{p}(e)}w_{k}^{\left(p\right)}(e)\;u_{k}^{\left(p\right)}(e)\right|}{\sum_{k\in \Omega_{p}(e)}w_{k}^{\left(p\right)}(e)+\varepsilon }.$$

Here, $u_{k}^{p}(e)$ denotes the unit phase vector for pixel e along path p, given by:

(20) $$u_{k}^{\left(p\right)}(e)=\frac{x_{k}^{\left(p\right)}(e)}{|x_{k}^{\left(p\right)}(e)|+\varepsilon }.$$

#### 2.4.3. Effective Number of Channels Factor $M_{eff}$

In practice, the number of reliable channels varies with spatial location. When only a few channels contribute, coherence estimates become more susceptible to random fluctuations. The $M_{eff}$ explicitly accounts for this evidence sufficiency and stabilizes the scoring across pixels. This parameter can be described as:

(21) $$M_{\mathrm{eff},p}(e)=\frac{{\left(\sum_{i}w_{i}\right)}^{2}}{\sum_{i}w_{i}^{2}}.$$

Notably, $M_{eff}$ summarizes the effective number of contributing channels (evidence sufficiency), whereas WPCF evaluates phase consistency after down-weighting unreliable channels; thus, they address channel participation from different perspectives and are complementary.

### 2.5. Defect-Indication Evaluation, Normal-Depth Assessment, and Quantitative Sizing

To ensure the objective, standardized, and consistent identification and quantitative evaluation of defect indications across different imaging results, a deterministic post-processing procedure was applied to each reconstructed amplitude image. The procedure comprises candidate-indication identification, SNR-based detectability evaluation, normal-depth localization, and quantitative sizing. In the present study, localization refers specifically to the normal-depth coordinate of an extracted indication.

Consistent with International Organization for Standardization (ISO) 16827:2025 [42], defect identification and sizing were treated as separate stages because discontinuity characterization and sizing are performed after an indication is detected. ISO 23865:2021 [43] provides general provisions for FMC/TFM inspection but does not prescribe universal acceptance levels for discontinuities. Accordingly, the −10 dB screening level, the −12 dB sizing level, and the associated image-processing parameters used in this study were applied consistently to all reconstructed images but were not treated as ISO acceptance criteria.

#### 2.5.1. Evaluation Gate and Normalized Response

The image amplitude and surface-normal depth at pixel e are denoted by I(e) and d(e), respectively. The evaluation was confined to the predefined valid depth gate. The $d_{min}$ and $d_{max}$ are defined according to the temporal widths of the front-surface and back-wall echo responses.

(22) $$\Omega_{g}=\left\{e|d_{min}\leq d(e)\leq d_{max}\right\}$$

The gate excludes the near-surface zone and the back-wall-dominated region from the evaluation domain; it does not by itself establish defect existence or size. Within this gate, the response was normalized as:

(23) $$R_{g}(e)=20\;\log_{10}\left[\frac{I(e)}{\max_{q\in \Omega_{g}}\;I(q)}\right]$$

Candidate pixels satisfying *R*_g_(*e*) ≥ −10 dB were used only to seed possible indication regions. The candidate pixels were grouped by eight-neighbor connectivity; components with a lateral extent below 0.2 mm were removed, and components separated by less than 1.0 mm in both coordinate directions were merged. Thus, the −10 dB level is a candidate-peak screening parameter rather than the boundary used for defect sizing.

#### 2.5.2. Objective Detectability Metric

For each retained candidate k, the local response peak is denoted by *I*_max,k_, and *I*_noise,k_ is the maximum amplitude in the predefined local high-noise comparison region. Defect detectability was quantified by:

(24) $$R_{g}(e)=20\;\log_{10}\left[\frac{I(e)}{\max_{q\in \Omega_{g}}\;I(q)}\right]$$

Using the maximum, rather than the mean, noise response provides a conservative and reproducible contrast metric. SNR is reported as an objective image-based detectability metric; no universal ISO acceptance threshold is claimed for the present specimen.

(25) $$R_{k}^{-12}={\mathrm{CC}}_{e_{\max,k}}\left\{e\in \Omega_{g}\;|20\;\log_{10}\left[\frac{I(e)}{I_{\max,k}}\right]\geq -12\right\}$$

where CC denotes the connected component containing the local peak. This −12 dB connected region is used for quantitative characterization and is not an independent defect-detection threshold. Its centroid provides the reported surface-normal depth, and the depth error is evaluated against the X-CT reference depth ($d_{CT}$) as:

(26) $$d_{c,k}=\frac{1}{\left|R_{k}^{-12}\right|}\sum_{e\in R_{k}^{-12}}d(e),\;e_{d,k}=|d_{c,k}-d_{\mathrm{CT}}|$$

Only the surface-normal depth difference is evaluated; neither a circumferential-position error nor a two-dimensional Euclidean localization error is reported.

#### 2.5.3. Principal Axis Sizing

The first principal axis is obtained from the coordinate covariance matrix of the −12 dB connected region. If ξ_k_(e) is the coordinate of pixel e projected onto this axis, the indication length and its relative error with respect to X-CT are:

(27) $$L_{k}=\max_{e\in R_{k}^{-12}}\;\xi_{k}(e)-\min_{e\in R_{k}^{-12}}\;\xi_{k}(e),\;\varepsilon_{L,k}=\frac{\left|L_{k}-L_{\mathrm{CT},k}\right|}{L_{\mathrm{CT},k}}\times 100\%$$

The amplitude-drop sizing principle follows ISO 16827:2025 [42], which addresses the characterization and sizing of already detected discontinuities. The −10 dB screening level, the −12 dB sizing level, the connectivity and merging rules, the centroid-based X-CT depth validation, and the principal axis projection are study-specific implementation choices and are not presented as ISO acceptance criteria.

## 3. Sample Preparation and Experimental Configuration

**Figure 5:**
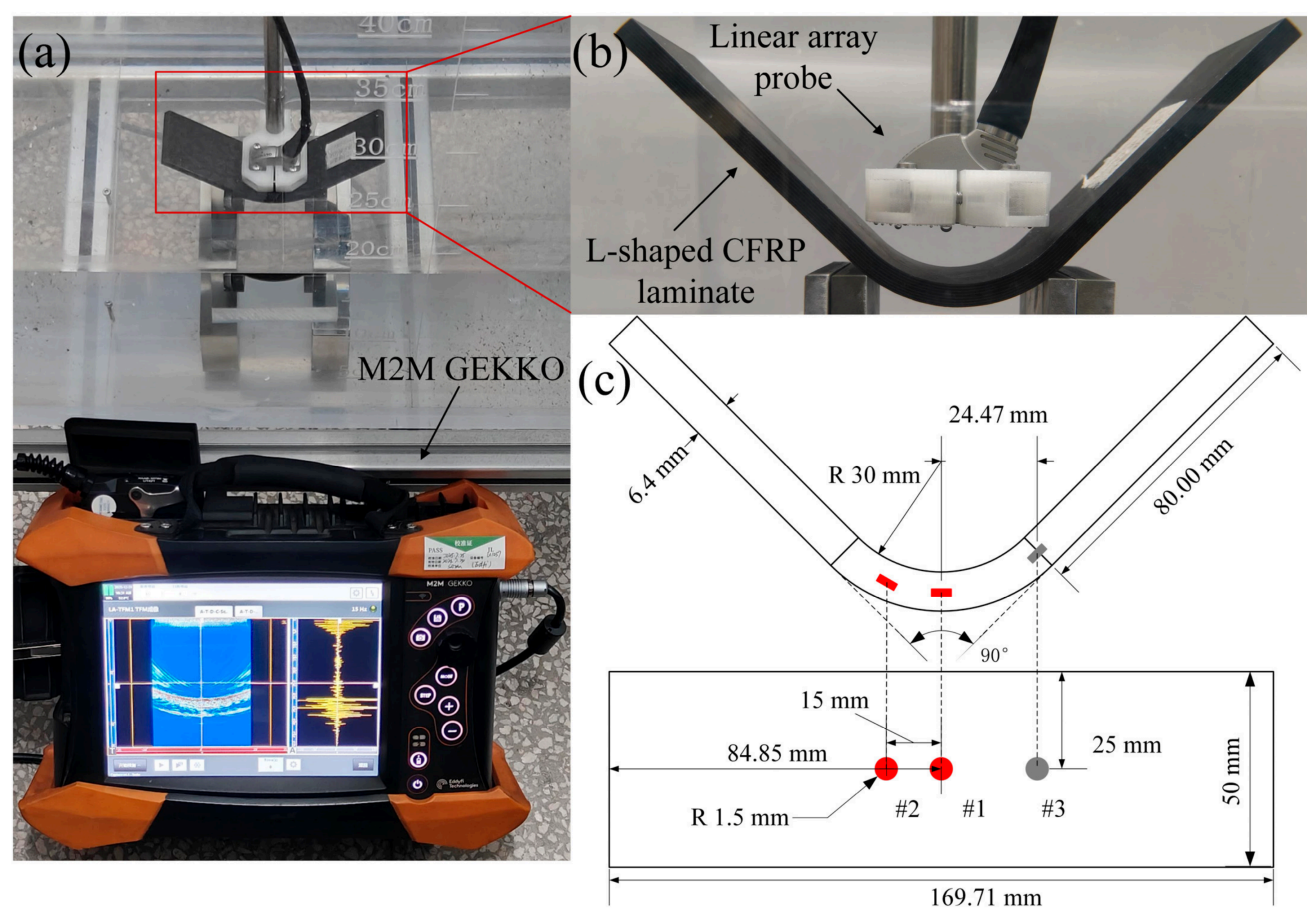
Data acquisition system and L-shaped CFRP specimen: (**a**) experimental configuration for ultrasonic data acquisition using FMC; (**b**) photograph of the experimental setup; (**c**) geometrical diagram of the L-shaped CFRP specimen with three embedded PTFE inserts. Defects #1 and #2, shown in red, are located within the curved corner region covered by the effective concave-side FMC field of view and were selected as the targets for ultrasonic imaging and quantitative evaluation. Defect #3, shown in gray, is located in the flatter arm region outside the evaluated FMC inspection range and was not included in the ultrasonic imaging analysis; it is retained in the schematic only to document the complete specimen design and embedded-insert configuration.

The experimental configuration and the geometric schematic of the L-shaped CFRP specimen are shown in Figure 5. In particular, Figure 5a presents the measurement system comprising the data acquisition unit, the water-immersion ultrasonic scanning apparatus, and the specimen under inspection. A 5L64-A32 linear array transducer (manufactured by Olympus Canada Inc., Richmond Hill, ON, Canada) was mounted on a fixed-orientation three-axis ultrasonic scanning system providing independent translations along the x-, y-, and z-directions, with a minimum selectable jogging increment of 0.01 mm. The probe orientation relative to the specimen was maintained by the mounting fixture. The specifications of the linear array transducer are summarized in Table 1.

The L-shaped CFRP laminate was constructed from 32 plies of T700 carbon fiber/epoxy unidirectional prepreg, which were stacked according to the layup sequence [45/0/-45/90]_4_[90/-45/0/45]_4_ and subsequently consolidated by hot-press curing. The thickness of each individual ply is approximately 0.2 mm. In the 0° ply, the fibers are arranged horizontally, and material properties are listed in Table 2. As shown in Figure 5c, the dimensions of the corner part are 6.4 mm-thick with a 90-degree angle. To simulate delamination defects, three polytetrafluoroethylene (PTFE) inserts with a diameter of Ф 3 mm were embedded at the 16th ply within the corner part. Defects #1 and #2 were located within the curved corner region covered by the concave-side FMC inspection and were selected for ultrasonic imaging and quantitative evaluation. Defect #3 was located in the adjacent flatter arm region rather than the corner region and was therefore not considered an inspection target; it is retained in Figure 5c only to document the complete specimen design.

**Figure 6:**
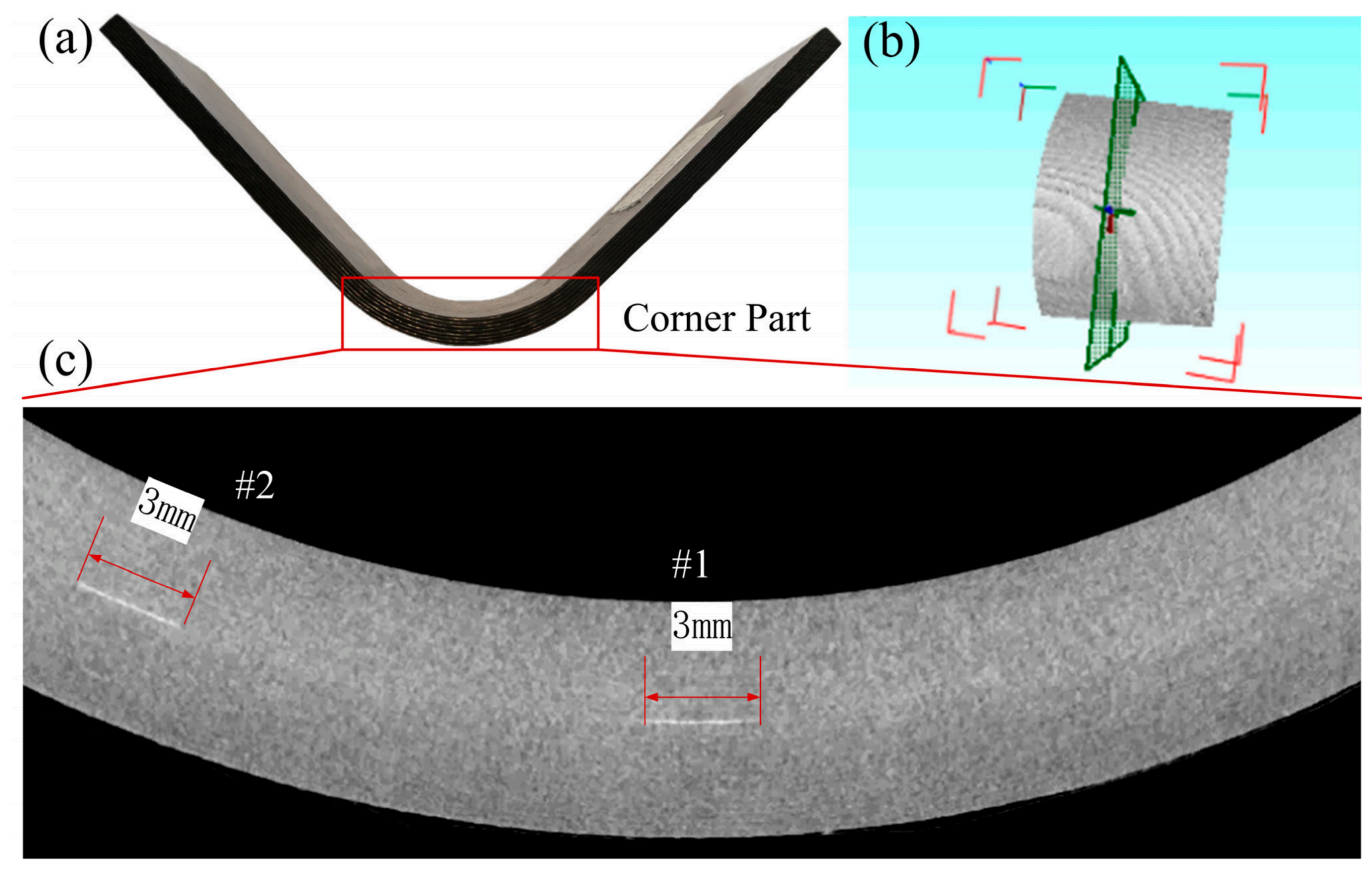
X-ray computed tomography (CT) reference results: (**a**) Specimen overview and corner part region indication; (**b**) CT three-dimensional (3D) reconstruction and extracted slice location schematic. The green plane indicates the CT cross-sectional view; (**c**) CT cross-sectional view passing through the centers of Defects #1 and #2 in the corner region inspected by concave-side FMC, where the circular inserts exhibit their maximum diameter of 3 mm. The X-CT results confirmed that the actual embedded positions were consistent with the specimen design drawing. Defects #1 and #2 were used for quantitative evaluation. Defect #3 is located in the adjacent flat region and was not an inspection target in this study; the complete specimen configuration is shown in Figure 5c.

For the present FMC inspections, the fixed-orientation probe was fully immersed in water and translated to the calibrated positions for Defects #1 and #2. Before acquisition, the scanning system coordinate frame was referenced to the geometric datum of the specimen. The insert-center positions were obtained from the specimen design drawing and confirmed by X-CT, which also verified the 3 mm reference diameters. Based on these X-CT-confirmed coordinates, the probe was positioned such that the two-dimensional B-scan plane passed through the center of each circular insert, while translation along the z-direction controlled the probe standoff and water path. This center-section positioning was used only for the controlled sizing validation and was not required as an input to the PCA-MPF-TFM reconstruction. Stable coupling between the probe and the specimen was provided by the water path, as shown in Figure 5b. A linear array probe was driven by an M2M GEKKO instrument (M2M, Les Ulis, France). Full matrix capture (FMC) was employed for excitation and reception, and time-domain signals of 64 × 64 channels were acquired at a sampling frequency of 100 MHz. The acquired data were then imported into in-house Python 3.11.13 code for post-processing and imaging. X-CT was therefore used as an independent reference for the insert positions and diameters, as shown in Figure 6.

The X-CT results confirmed that the actual embedded positions of Defects #1 and #2 were consistent with their designed locations. The X-CT-confirmed center positions were subsequently used to define the calibrated B-scan positions for diameter sizing.

## 4. Results and Discussion

### 4.1. Group Velocity Profile

**Figure 7:**
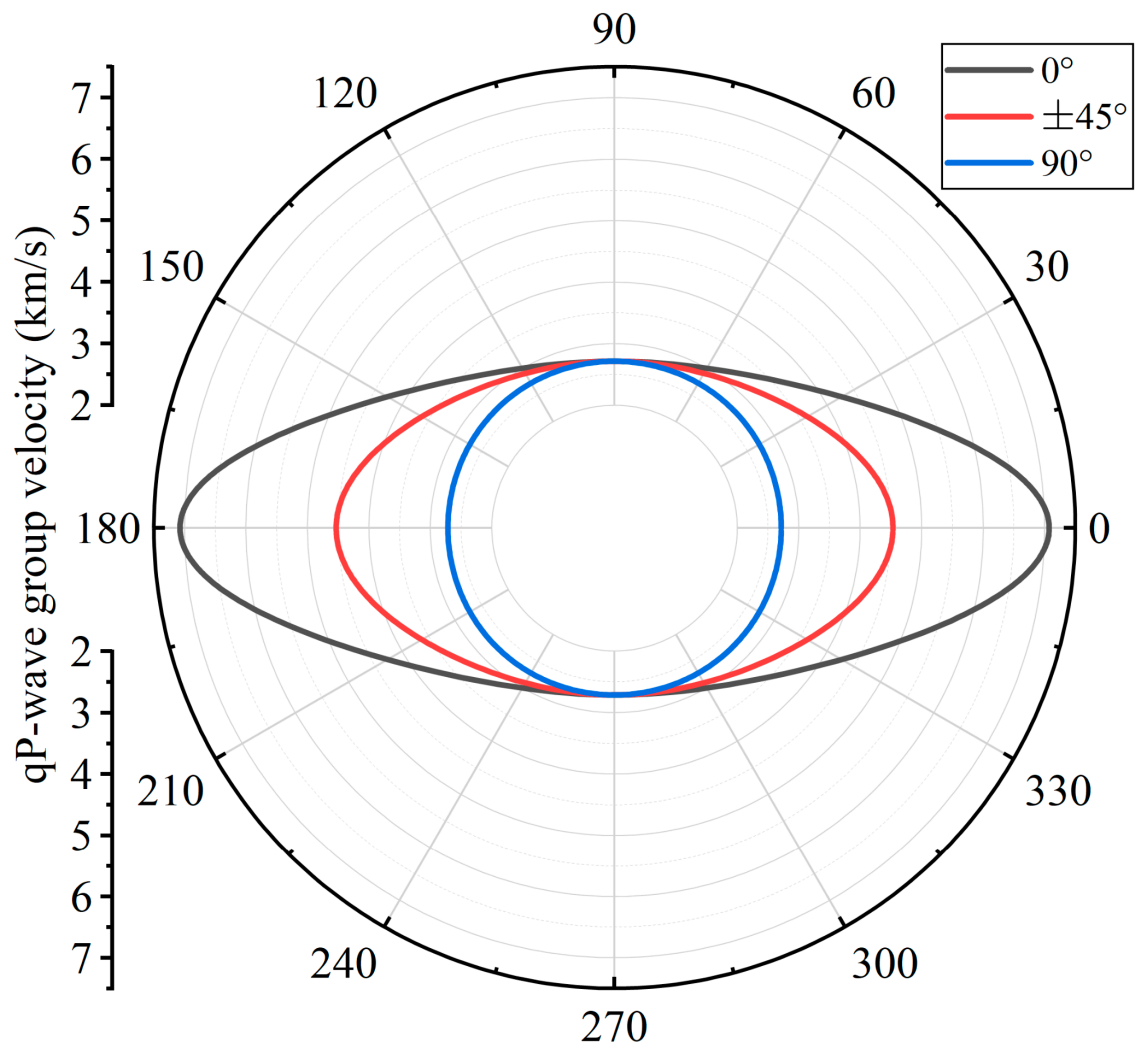
Variation in qP-wave group velocity with propagation angle in 0°, ±45° and 90° unidirectional CFRP plies.

Based on the single-ply material properties of CFRP listed in Table 2, five independent elastic constants are obtained and substituted into (1). Furthermore, for plies with zero degree, plus and minus 45 degrees, and 90 degrees in L-shaped CFRP, in-plane quasi-longitudinal wave group velocity is calculated using the method described above. Variation in in-plane group velocity with propagation angle is shown in Figure 7.

### 4.2. Imaging Results of Total Focusing Method Based on Weak-Refraction Path and Shortest Path

To evaluate the influence of path assumptions on focusing quality, Following Section 2, two candidate paths are constructed for each pixel: the minimum-time (min-time) path and the weak-refraction (WR) path [44]. Unless otherwise stated, every image and quantitative result reported in Chapter 4 was assessed using the single deterministic procedure defined in Section 2.5. The displayed dynamic range is used only for visualization and does not alter indication identification, SNR evaluation, localization, or sizing.

**Figure 8:**
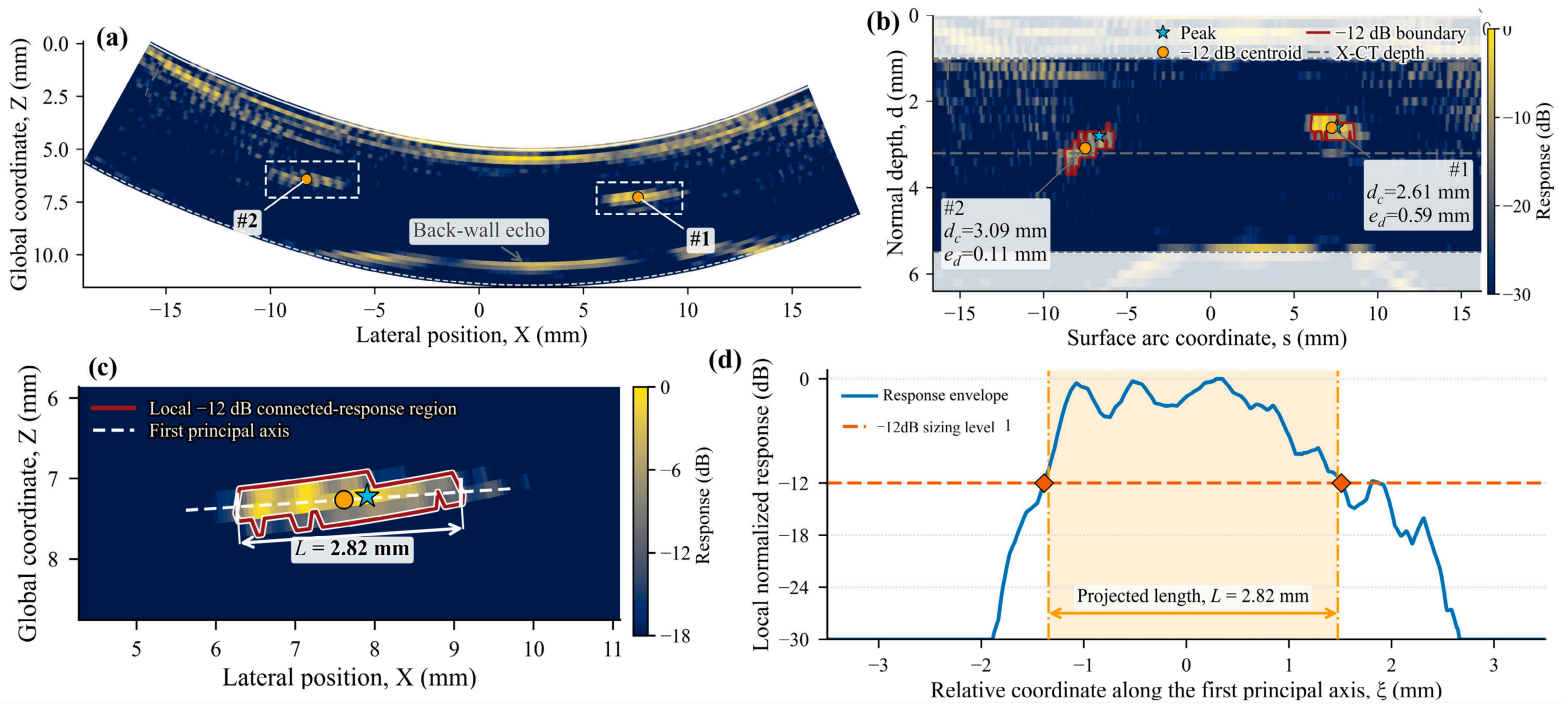
Illustration of the defect localization and quantitative sizing workflow using the PCA-MPF-TFM imaging result as an example. (**a**) Identification of defect indications in the final image. (**b**) Transformation to the surface-arc/normal-depth coordinate system and determination of the indication positions. (**c**) Extraction of the connected −12 dB indication region and determination of the principal measurement direction using PCA. (**d**) Amplitude-drop sizing along the principal axis, with the −12 dB crossings defining the indication endpoints and apparent length $L_{PCA}$. Defect sizing based on the −12 dB drop technique specified in ISO 16827:2025.

Figure 8 illustrates this unified procedure using the PCA-MPF-TFM reconstruction as a representative example. Evaluation is first restricted to the valid surface-normal depth gate, after which the gated response is normalized. Candidate indications are seeded at −10 dB, grouped by eight-neighbor connectivity, filtered using the 0.2 mm minimum lateral extent, and merged when their separations in both coordinate directions are below 1.0 mm. For each retained indication, detectability is quantified by the peak-to-local-maximum-noise SNR defined in Section 2.5; the connected −12 dB region containing the local peak is then used for centroid-based normal-depth localization and principal axis sizing, with the corresponding errors evaluated against the X-CT reference. The same gate, thresholds, connectivity and merging rules, noise-region definition, and reference values were applied to every reconstruction.

Specifically, Figure 8a shows the candidate-indication and local high-noise regions used for detectability evaluation; Figure 8b shows the surface-arc/normal-depth transformation and the centroid used to report the indication depth; Figure 8c shows the connected −12 dB region and the principal axis determined by principal component analysis (PCA); and Figure 8d shows the amplitude profile along that axis, whose two −12 dB crossings define the apparent length. The −10 dB level is used only to screen candidate peaks, whereas the −12 dB connected region is used only after detection for localization and sizing; neither level is interpreted as an ISO acceptance threshold.

For all reconstructions, the water-path sound speed was set to 1500 m/s, the discretization step to 0.05 mm, and the specimen’s curved surface was represented by the following polynomial:

(28) $$y=597.7x^{4}+0.4017x^{3}-16.66x^{2}+0.076x+17.59e^{-2}.$$

**Figure 9:**
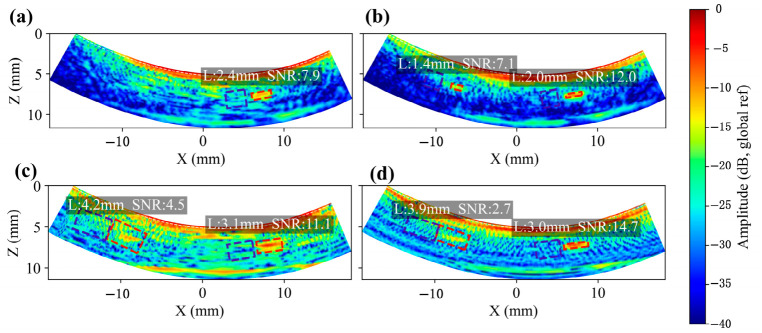
B-scan images of the corner part produced by standard TFM using ray paths with (**a**) TFM; (**b**) TFM with weak reflection ray path; (**c**) ray tracing total focusing method (RT-TFM) with minimum travel time; (**d**) RT-TFM with weak reflection ray path, both (**a**,**b**) isotropic and (**c**,**d**) anisotropic cases.

**Figure 10:**
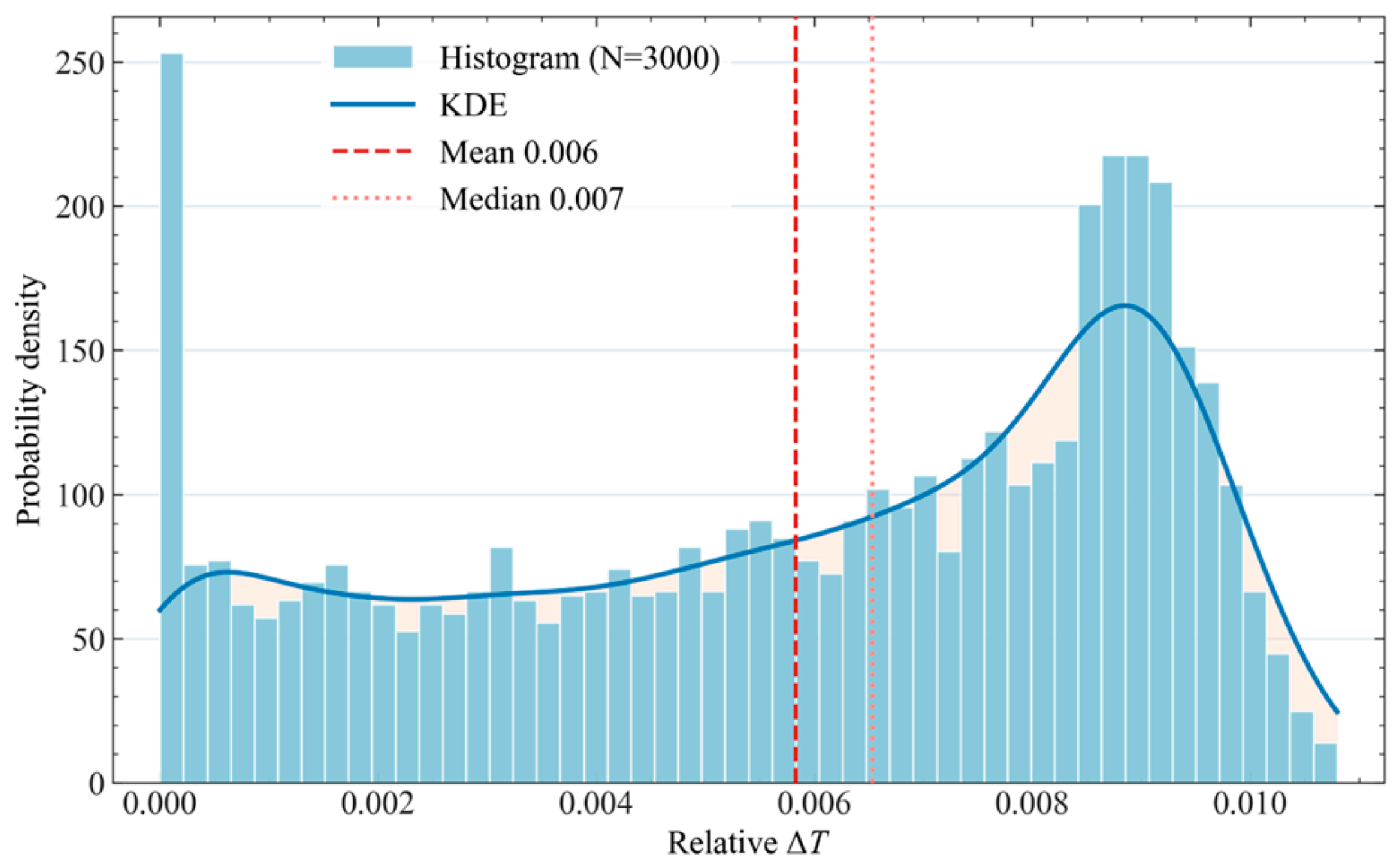
Statistical plot of the travel time differences between the weak-refraction path and the minimum travel time path.

(1) Results under isotropic delay modeling: In the isotropic modeling case, the standard TFM in Figure 9a forms a visible indication at defect #1, yet with broadened and locally blurred boundaries, implying residual delay mismatch. For defect #2, pronounced stripe-like coherent artifacts emerge near the corner and partially mask the defect. With the WR ray path (Figure 9b), the separability of defect #2 improves, suggesting that WR can suppress interface-coupled echoes associated with strongly bent paths; however, the effective aperture may be reduced, leading to underestimated −12 dB lengths. Overall, the images highlight a key trade-off: WR tends to reduce coherent artifacts but may introduce sizing bias.

(2) Comparison after anisotropic travel time correction: the min-time-corrected reconstruction (RT-TFM) in Figure 9c shows a marked improvement for defect #1, providing a 3.2 dB SNR increase over standard TFM and reducing the −12 dB length error to 0.1%, indicating good compatibility with the min-time hypothesis when anisotropy is accounted for. Defect #2 remains limited by persistent stripe-like coherent clutter that elevates the local noise floor and biases threshold-based sizing; the banded, repeatable morphology suggests deterministic multi-interface reverberations that can still be partially phase-aligned by the delay model. For weak-reflection ray tracing TFM (WR-RT-TFM; Figure 9d), the left-side response is comparable to the stripes (SNR 2.7 dB), whereas the right-side response becomes more concentrated (SNR ≈ 14.7 dB), implying improved local delay alignment and partial stripe suppression. Hence, the two path assumptions exhibit spatially complementary validity, and a single-path model is unlikely to simultaneously ensure detectability and quantitative stability across the full field of view.

(3) Quantifying the non-equivalence of the two path hypotheses: To quantify the discrepancy between the two hypotheses, 3000 element–pixel pairs are randomly sampled and the relative travel time difference is computed as

(29) $$\Delta T=\frac{\left|t_{ij}^{\left(\mathrm{wr}\right)}(e)-t_{ij}^{\left(\mathrm{sp}\right)}(e)\right|}{t_{ij}^{\left(\mathrm{sp}\right)}(e)}.$$

where $t_{ij}^{\left(wr\right)}\left(e\right)$ and $t_{ij}^{\left(sp\right)}\left(e\right)$ denote the weak-refraction path and min-time travel times, respectively. As shown in Figure 10, exhibits a clear mixed distribution: a sharp spike near Δ≈0 indicates a subset of geometries where both hypotheses coincide, whereas the dominant mass concentrates around a non-zero band (with a major peak around 0.008–0.009).

This confirms that the WR and min-time hypotheses are not interchangeable as equivalent delay models in corner inspections; rather, they represent two competing local approximations whose validity varies spatially.

The above results establish two key observations: (i) each single-hypothesis reconstruction can fail locally due to delay mismatch, and (ii) the remaining stripe-like artifacts are largely coherent clutter, which can be reinforced whenever delays accidentally provide phase alignment. These observations directly motivate a pixel-wise, data-driven mechanism to discriminate and/or fuse candidate paths based on phase consistency evidence, which is developed in the next subsection and later visualized through weight/coherence difference maps.

### 4.3. Imaging Results of Phase-Consistency-Adaptive Multi-Path Fusion and Performance Comparison

**Figure 11:**
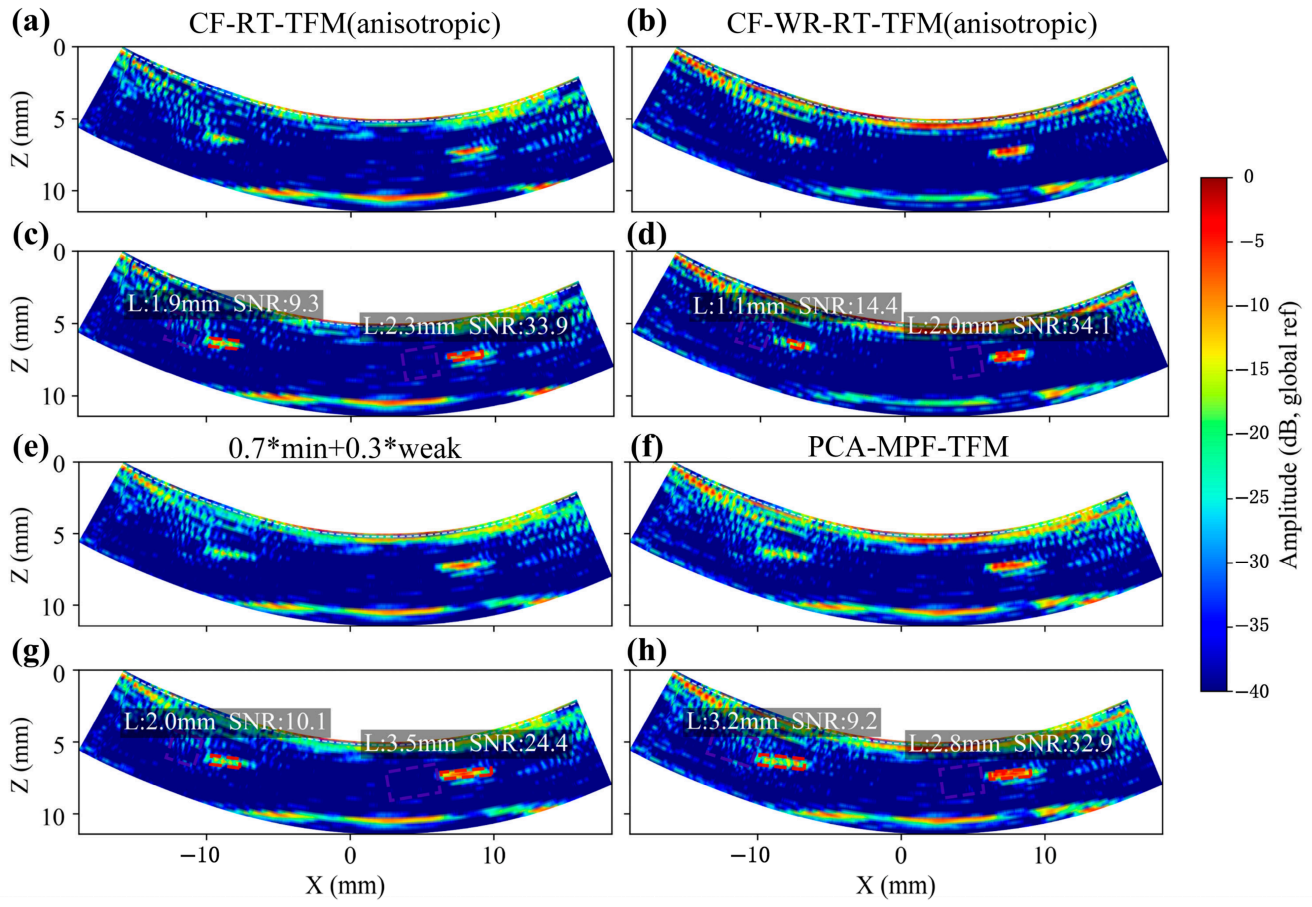
Comparison of anisotropic multi-path TFM imaging: (**a**,**c**) the coherence-factor-weighted ray tracing total focusing method (CF-RT-TFM) with minimum travel time; (**b**,**d**) the CF-RT-TFM with weak reflection travel time; (**e**,**g**) the multi-path fusion with fixed weights, and (**f**,**h**) the adaptive fusion (PCA-MPF-TFM).

**Figure 12:**
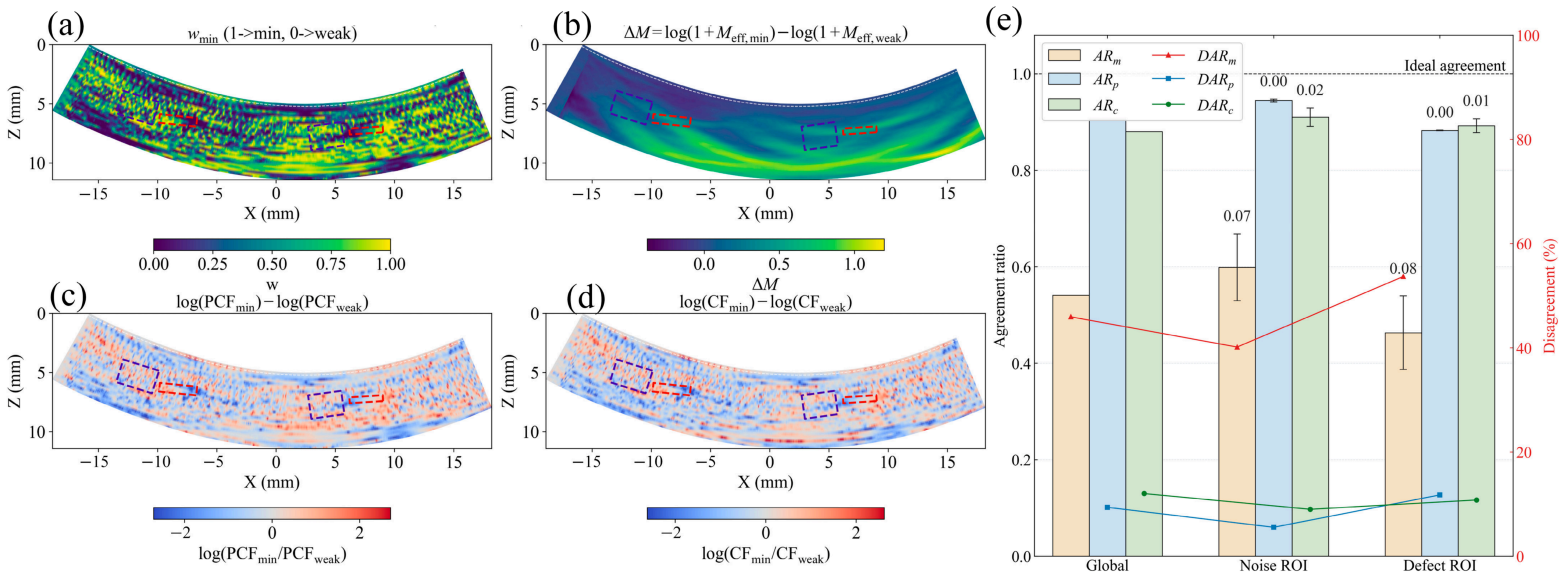
Phase-consistency-adaptive multi-path selection and agreement statistics: (**a**) per-pixel path selection weight map $w_{min}$; (**b**) effective-channel-count difference ΔM; (**c**) phase-coherence difference; (**d**) amplitude coherence difference; (**e**) agreement ratio statistics for global/noise ROI/defect ROI (with error bars and ideal-agreement reference). The region enclosed by the red dashed line is the defect ROI, while the region enclosed by the blue dashed line is the noise ROI.

**Figure 13:**
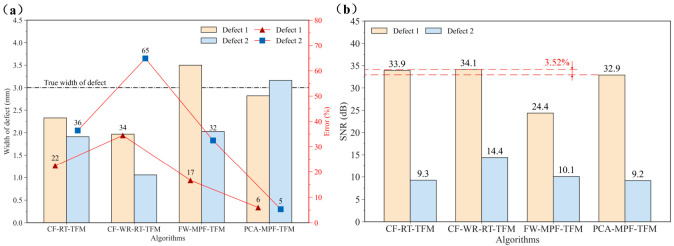
Quantitative comparison of two inspected defects using different TFM imaging algorithms: (**a**) measured defect width and relative error; (**b**) defect image SNR comparison.

To examine whether phase-consistency-driven fusion can translate the coexistence of multiple ray path solutions into more reliable imaging, this section compares four representative strategies under the anisotropic model [44,45,46,47]: (i) coherence-weighted single-path reconstructions using the minimum travel time hypothesis and the weak-refraction hypothesis, (ii) a global fixed-weight fusion, and (iii) the proposed pixel-wise adaptive fusion (PCA-MPF-TFM). Qualitative B-scan results are compared in Figure 11. Figure 12 presents the phase-consistency-adaptive multi-path selection and consistency statistics for all pixels in the image. All quantitative comparisons follow the Section 2.5 procedure illustrated in Figure 8: detectability is reported using the local maximum-noise SNR, localization uses the centroid of the connected −12 dB region, and sizing uses the PCA principal direction and the two −12 dB crossings. The resulting apparent lengths and relative length errors are shown in Figure 13a, the image SNR values in Figure 13b, and the detailed values in Table 3.

(1) Imaging results obtained using individual paths: Results of shortest-path travel time-corrected TFM with amplitude coherence weighting (CF-RT-TFM) are presented in Figure 11a,c. Stripe-like background noise is markedly suppressed, yet the defect boundary is also affected, leading to shrinkage of apparent defect size. For weak-refraction-based TFM with amplitude coherence weighting (CF-WR-RT-TFM), as shown in Figure 11b,d, background noise in the central region is further removed compared with CF-RT-TFM, thereby yielding evident improvement in SNR at the defect location. Meanwhile, while the weak-refraction path suppresses the background, local propagation model mismatch compresses the defect boundary under −12 dB drop sizing, resulting in further underestimation of defect length. These observations indicate that higher SNR is not equivalent to a more accurate delay model, and two path hypotheses already exhibit complementary local advantages.

(2) Global fixed-weight fusion fails to inherit local advantages consistently: A global empirical fusion, fixed-weight multi-path-fusion total focusing method (FW-MPF-TFM), is further tested in Figure 11e,g. The fusion does not consistently preserve the strengths of both hypotheses; instead, a pronounced SNR degradation is observed for defect #1, accompanied by an overestimation in length. This behavior is primarily attributed to local delay mismatch: once one candidate hypothesis becomes inaccurate at a given pixel, fixed-weight fusion forces both well-aligned and misaligned channels to be summed simultaneously, canceling coherent gain and spreading energy. The failure of fixed fusion provides direct evidence that effective true paths are distributed non-uniformly in space and cannot be represented by a single path set or a global mixing ratio.

(3) PCA-MPF-TFM improves sizing reliability while maintaining high SNR: The proposed PCA-MPF-TFM results in Figure 11f,h show continuous defect indications with clearer contours compared with single-path reconstructions and fixed fusion. A small amount of regular stripe-like residual artifacts remains, which mainly originates from deterministic coherent structural echoes. Such components can achieve stable phase alignment under both candidate delay hypotheses, yielding high scores for both paths and therefore being retained after fusion. Importantly, these residual components are largely separated from defect responses and thus exert limited influence on defect detection and sizing evaluation. Quantitatively, Figure 13 shows that PCA-MPF-TFM substantially reduces −12 dB drop sizing errors while keeping SNR at a competitive level; detailed values are summarized in Table 3. Overall, compared with RT-TFM, the proposed method achieves a notable SNR improvement and reduces average length estimation error, and further decreases the sizing bias relative to coherence-denoised single-path baselines (CF-RT-TFM and CF-WR-RT-TFM).

(4) To validate the rationality of the pixel-wise adaptive fusion strategy proposed in Section 2.4: Figure 12 further presents path-weight map and agreement ratio (AR) statistics. As shown in Figure 12a, path weight $w_{min}$ exhibits a pronounced spatially non-uniform distribution. Within defect regions of interest (ROIs) marked by red boxes, $w_{min}$ allows each defect region to be roughly divided into two triangular subregions, where parts with $w_{min}\approx 0$ are consistently located closer to the imaging-region center. This indicates that the weak-refraction path tends to identify the central area, where the refraction effect is weaker, as a defect-related region; the shortest path tends to attribute the circumferential area with shorter travel time to the defect region. Correspondingly, spatial maps of phase-coherence difference $log({PCF}_{min})-log({PCF}_{weak})$ and amplitude coherence difference $log({CF}_{min})-log({CF}_{weak})$ in Figure 12c,d reveal the same trend. Such behavior supports the viewpoint that shortest-path set $P_{min}$ and weak-refraction set $P_{weak}$ are two candidate subsets of true propagation-path set $p^{*}(e)$. It also partially explains the evident underestimation of defect size observed in Figure 11 for CF-RT-TFM and CF-WR-RT-TFM reconstructions.

By contrast, ΔM in Figure 12b is relatively uniform and is better interpreted as an evidence-sufficiency term rather than a physical-path selector. When ΔM>0, more effective channels support the min-time delays, stabilizing the decision; when ΔM ≈ 0, both hypotheses are supported by comparable evidence and the weight becomes more reliant on coherence contrasts, especially in weak-echo regions. Consistently, the AR of $M_{eff}$ stays near 0.5 across the global/noise/defect ROIs, whereas the ARs of phase- and amplitude coherence criteria remain high (>0.85) (Figure 12e). Overall, ΔM mainly regularizes the weight estimation against channel-amplitude fluctuations, while the final fusion weights are dominated by coherence contrasts, enabling robust ROI-invariant pixel-wise path switching.

These results indicate that the weight map in Figure 12a shows that optimal path selection exhibits pronounced spatial variation, directly corroborating the local switching behavior of the true propagation hypothesis. Meanwhile, phase-coherence and amplitude coherence differences in Figure 12c,d remain consistent with the weight distribution, and agreement ratio statistics in Figure 12e indicate that coherence-based criteria provide stable discriminative capability across different ROIs.

To verify that the reported sizing results were reproducible rather than incidental, three independent FMC acquisitions were performed. Before each repeated acquisition, the three-axis scanning system was returned to its reference position and subsequently translated to the same calibrated position corresponding to the X-CT-confirmed center section of each insert. The probe orientation, acquisition parameters, water-path condition, and processing parameters were maintained unchanged. No discernible difference in the estimated defect diameters was observed among the three acquisitions within the resolution of the present image-based sizing procedure. The estimated diameters remained approximately 2.82 mm for Defect #1 and 3.20 mm for Defect #2. Therefore, no clearly resolvable sizing variation was observed under the tested return-to-position condition.

The minimum selectable jogging increment of the scanning system was 0.01 mm, whereas the reconstruction grid increment was 0.05 mm. The lack of a discernible difference indicates that any sizing variation was below the resolution of the present image-based sizing procedure and should not be interpreted as zero physical measurement uncertainty.

## 5. Conclusions

This work addresses ultrasonic array inspection of L-shaped CFRP corner parts, where nonplanar interfaces lead to Snell law multi-solution propagation and hence ray path non-uniqueness. To cope with this challenge, a phase-consistency-driven adaptive multi-path fusion algorithm, termed PCA-MPF-TFM, is developed to improve focusing robustness while maintaining sizing accuracy. The three PTFE inserts are visible in the CT reference results. In the present FMC validation, Defects #1 and #2 were selected as the inspected targets, while Defect #3 was not included in the quantitative imaging analysis because it was outside the effective inspection region of the current FMC acquisition.

The results show that defects poorly resolved by isotropic TFM can be reliably detected. Compared with RT-TFM, the defect SNR improves by up to 21.8 dB and the average length-estimation error decreases by 15%. Relative to CF-RT-TFM and CF-WR-RT-TFM, the average length error is further reduced by 23.3% and 41.7%, respectively (Table 3). Overall, given known layup configurations, the proposed method offers a promising route for high-quality defect detection and quantitative characterization in CFRP corner regions.

All imaging algorithms in this work are deployed on a Linux platform. Single-source shortest path search and multi-source shortest path search based on Dijkstra’s algorithm are accelerated on an RTX 4060 Laptop graphics processing unit (GPU) via the RAPIDS 25.08 computing library. For a model with 21,454 nodes and 5,813,272 valid edges, full PCA-MPF-TFM achieves an average runtime of 127.03 s, where shortest-path search consumes 12.89 s on average.

## Figures and Tables

**Table 1:** Specifications of the 5L64-A32 linear array probe.

Specifications	Value
Center frequency	5 MHz
Elements	64
Array Element Spacing	0.5 mm
Array Element Width	0.4 mm
Activate Aperture	32 mm

**Table 2:** Material properties of T700/epoxy composites.

Sample	L-Shaped CFRP Laminate
Density	1.512 g/cm^3^
Longitudinal Young’s modulus (E1)	71.466 GPa
Transverse Young’s modulus (E2)	8.592 GPa
Longitudinal–transverse shear modulus (G12)	5.740 GPa
Transverse–transverse shear modulus (G23)	3.059 GPa
Major Poisson’s ratio ($\nu_{12})$	0.4655
Transverse Poisson’s ratio ($\nu_{23})$	0.4519

**Table 3:** Algorithm performance comparison. (Values in Table 3 are rounded to one decimal place).

Algorithms		Isotropic Model		Anisotropic Model					
		TFM	WR-TFM	RT-TFM	WR-RT-TFM	CF-RT-TFM	CF-WR-RT-TFM	FW-MPF-TFM	PCA-MPF-TFM
#1	Length (mm)	2.4	2.0	3.1	3.0	2.3	2.0	3.5	2.8
	SNR (dB)	7.9	12.0	11.1	14.7	33.9	34.1	24.4	32.9
#2	Length (mm)	/	1.4	4.2	3.9	1.9	1.1	2.0	3.2
	SNR (dB)	/	7.1	4.5	2.7	9.3	14.4	10.1	9.2

## Data Availability

The original contributions presented in this study are included in the article. Further inquiries can be directed to the corresponding author.
